# Strategic Health Communication as a Value Practice: Insights From the German‐Speaking Healthcare Systems

**DOI:** 10.1002/hsr2.73491

**Published:** 2026-11-05

**Authors:** Slavko Rogan, Katja Schlegel, Eefje Luijckx

**Affiliations:** ^1^ School of Health Professions, Division of Physiotherapy Bern University of Applied Sciences Bern Switzerland; ^2^ Faculty of Human Science, Institute of Psychology University of Bern Bern Switzerland

**Keywords:** health care surveys, health communication, health literacy, public relations

## Abstract

**Background and Aim:**

This study synthesizes value‑related patterns of strategic health communication across macro, meso and micro levels and analyses their relevance for functional, interactive, critical, digital and organizational health literacy (HL).

**Methods:**

A narrative, theory‑informed synthesis was conducted, integrating 15 empirical and conceptual articles from Austria, Germany and Switzerland. Evidence from population surveys, organizational assessments, digital content analyses, qualitative youth studies and crisis‑communication research was categorized across system levels and analysed for explicit and implicit value dimensions.

**Results:**

Five value configurations emerged across contexts: clarity–safety–rights, participation–appreciation–co‑production, trust–transparency–consistency, inclusion–equity–accessibility and digitality–risk literacy–source criticism. Structural obstacles such as low readability of health websites, organizational gaps in patient involvement and marked digital HL inequalities among adolescents and multimorbid older adults were consistently documented.

**Discussion:**

Strategic health communication succeeds when systemic, organizational and interpersonal values align. Misalignments create HL gradients, while value‑driven practices such as plain language, teach‑back, multimodal formats and empathetic crisis communication enhance comprehension, trust and engagement. Value‑oriented communication frameworks are therefore essential for equitable HL promotion.

## 1 Introduction

Strategic health communication extends beyond information transmission. Strategic health communication refers to the deliberate planning, implementation, and evaluation of communication activities intended to achieve health‐related objectives. It encompasses communication conducted by governments, public health agencies, healthcare organizations, and health professionals across policy, organizational, and interpersonal contexts. From this perspective, strategic health communication operates at macro‐, meso‐, and micro‐levels and contributes to shaping how individuals' access, understand, appraise, and use health information and services.

It shapes meaning, negotiates legitimacy, and reflects the values embedded in institutions, professional routines, and public expectations. Within this context, health literacy (HL) has emerged as a key concept linking communication, equity, and participation. Contemporary health literacy frameworks increasingly emphasize active engagement in health‐related decision‐making, navigation of health systems, and participation in healthcare processes as important outcomes of health literacy [1, 2, 3]. In its international definition, HL encompasses the knowledge, motivation, and competencies needed to access, understand, evaluate, and apply information in contexts of care, prevention, and health promotion [4]. More recently, HL has also been described as people's ability to access, understand, appraise, and use health information and services in ways that promote and maintain good health and well‐being. From this perspective, strategic health communication can be understood as a key mechanism for enabling health literacy by shaping the conditions under which individuals interact with health information and services. This definition has informed a wide range of measurement approaches, including the European Health Literacy Survey (HLS‐EU), as well as policy development and implementation strategies across Europe [5]. Over time, the field has expanded to include instruments targeting specific dimensions of health literacy, including organizational health literacy and health‐literate healthcare organizations [1, 6, 7].

Over the past decade, HL has shifted from a primarily individual skill set to a multidimensional construct spanning individual, organizational, and systemic levels. The *International Handbook of Health Literacy* documents this conceptual expansion, emphasizing relational, participatory, and context‐sensitive perspectives that integrate empowerment and inclusion [5]. Recent German‐language scholarship highlights the alignment between societal demands and available resources, particularly in digital information environments and during crises [8, 9].

Empirical evidence from Germany underscores the relevance of this broader view. Representative national data reveal persistently limited HL in large segments of the population and substantial deficits in digital and navigational HL, as documented in the HLS‐Germany (HLS‐GER 3) report [9, 10, 11]. Similar disparities appear at the subnational level: in the Canton of Zurich (Switzerland), HL is associated with socioeconomic position, and more than half of respondents report difficulties with basic processes such as accessing, understanding, evaluating, and applying health information [9, 12].

At the same time, digital transformation amplifies tensions between normative expectations and real‐world communication conditions. A large‐scale analysis of German‐language health websites from Germany, Austria, and Switzerland (referred to by the authors as the German Health Web) showed that many websites exhibit poor readability despite using largely correct medical terminology [13]. Complementary Swiss studies reveal systematic associations between HL and trust: individuals with higher HL report greater trust in authorities and health professionals, whereas social media consistently receive lower trust ratings [14]. These findings highlight tensions between credibility, comprehensibility, and digital reach that contemporary strategic communication must navigate.

In German‐speaking healthcare systems, health literacy research has evolved into a distinct field characterized by strong links between population health literacy measurement, organizational health literacy, and health policy initiatives. Foundational scholarship has contributed to a broader understanding of health literacy as an interaction between individual competencies, organizational responsibilities, and system‐level conditions [15, 16]. This perspective has supported the development of organizational and system‐oriented approaches that move beyond individual deficit models and emphasize accessibility, participation, and equity in healthcare delivery [1, 2]. These conceptual developments have also informed national and regional health literacy strategies across Germany, Austria, and the German‐speaking regions of Switzerland, where communication quality, trust, and digital transformation have become central policy priorities [3, 17].

At the macro level, governmental communication and evidence‐informed policy advice operate in an environment shaped by politicization, reputation management, and trust building. Research on the German Federal Institute for Risk Assessment illustrates how complex risk topics, the emergence of new actors including influencers, and disinformation challenge institutional values such as objectivity, legitimacy, and participation. At the same time, studies show that visualization, stakeholder involvement, and participatory formats serve as instruments to stabilize institutional legitimacy [13, 18].

At the meso level, organizational health literacy (OHL) has become a strategic framework for improving communication environments. OHL emphasizes the responsibility of organizations to provide information and services in ways that allow users to find, understand, evaluate, and apply information with minimal barriers. International frameworks have been adapted into measurable standards and tools, including instruments from the World Health Organization (WHO) Action Network on Measuring Population and Organizational HL (M‐POHL) [6, 19]. Established instruments include the Health‐Literate Health Care Organization 10‐item Questionnaire (HLHO‐10), which operationalizes key organizational attributes such as leadership commitment, communication quality, navigation support, and patient involvement, as well as patient‐reported measures such as the Health Literacy‐Sensitive Communication Scale (HL‐COM) [20, 21].

Recent empirical studies in Germany report moderate to good self‐assessments but highlight deficits in patient‐centred communication, multimodal information provision, and participation structures [7, 22]. Evidence from primary care settings underlines the importance of communication and information coordination at care interfaces, while pilot projects show that sustainable improvements in patient outcomes require structural embedding rather than isolated interventions [22]. For vulnerable groups, such as people with disabilities, research demonstrates the need for adapted communication formats, accessible structures, and co‐creation processes [23]. Overall, current scholarship positions organizational HL as a key component of quality development and equity in care [1].

At the micro level, relationship quality, trust, involvement, and emotional safety emerge as central communicative values. Qualitative findings from Switzerland show how adolescents draw on family, peers, teachers, health professionals, and digital sources, and how accessibility, rapport, and opportunities for participation shape their health literacy practices [24]. Representative German data reveal substantial social and age related inequalities in digital health literacy among children and adolescents, particularly in search skills, evaluation of reliability, and privacy protection [25].

Contemporary health literacy research increasingly conceptualizes health literacy as a multi‐level phenomenon that emerges from interactions between system‐level conditions, organizational environments, and individual capabilities [1, 2, 3]. Within these frameworks, communication is not merely the transmission of information but a mechanism through which normative values such as transparency, participation, equity, accountability, trust, and person‐centredness are enacted across policy, organizational, and interpersonal contexts. These values are frequently referenced in health literacy and organizational health literacy frameworks; however, their role as explicit analytical categories has rarely been examined systematically.

Although health literacy has increasingly been conceptualized across macro‐, meso‐, and micro‐level dimensions, several gaps remain [1, 2, 3]. First, it often remains unclear which normative orientations guide communication in policy, organizations, and interpersonal interactions. Second, there is no systematic analysis of how values such as transparency, participation, fairness, trustworthiness, or user orientation become visible in communication processes and how they relate to different forms of health literacy. Third, little is known about the extent to which institutionally formulated values, such as mission statements, strategies, or quality standards, align with or diverge from values enacted in everyday communication practices.

In this contribution, values are understood as stable normative principles that shape, legitimize, or constrain communication at governmental, organizational, and interpersonal levels. In international health literacy discourse, values often appear indirectly through concepts such as equity, empowerment, inclusion, accountability, or person‐centeredness. However, an explicit and systematic analysis of values within health literacy research remains largely absent. This gap provides the opportunity to reconsider the relationship between strategic health communication and health literacy from a value‐oriented perspective.

This contribution therefore aims to identify value‐related patterns in strategic health communication across macro‐, meso‐, and micro‐levels and to analyse how these patterns support or hinder individuals' ability to access, understand, appraise, and use health information and services, thereby influencing different forms of health literacy, including functional, interactive, critical, digital, and organizational health literacy. Particular attention is given to the tensions between articulated and enacted values across policy communication, organizational structures, and interpersonal interactions. On this basis, the study provides a scientific foundation for value‐oriented communication strategies that foster fairer information environments, more resilient organizations, and a public communication landscape that strengthens health literacy within German‐speaking healthcare systems.

## 2 Methods

### 2.1 Study Design

This study was designed as a narrative, theory‑driven synthesis to map value‑related patterns of strategic health communication across macro, meso, and micro levels in the German‑speaking context. This design is particularly suitable for research fields characterized by heterogeneous evidence, complex conceptual developments, and rapidly evolving contexts, where a narrative approach enables the integration of empirical findings, theoretical perspectives, and contextual influences [26]. The objective was not to achieve exhaustive coverage of all publications but rather to conduct a theoretically informed selection of empirical and conceptual contributions that explicitly or implicitly address values and demonstrate relevance for health literacy, including its digital and organizational dimensions. The synthesis integrates quantitative population studies, regional surveys, organizational investigations, digital content analyses, qualitative studies with adolescents, and conceptual contributions to the development of the health literacy construct.

### 2.2 Narrative Search Strategy

As the aim of this study was not comprehensive evidence aggregation but a theory‑informed synthesis, the search strategy combined structured database searches with free‑text searches, backward citation tracking, and theoretical sampling of conceptual literature. The approach followed established principles for narrative reviews as outlined by Greenhalgh et al. [26] and incorporated controlled vocabulary such as MeSH terms together with keywords, synonyms, and context‑specific terminology relevant to value‑based strategic health communication in German‑speaking healthcare systems.

Literature identification was iterative and proceeded in several steps. First, a structured search was conducted in CINAHL, Embase and PubMed using controlled vocabulary and free‑text terms. The principal search concepts were health literacy, strategic communication, values (e.g., transparency, trust, participation, and equity), organizational health literacy, and the German‐speaking healthcare context. To enhance methodological transparency, an overview of the search domains, illustrative search terms, and data sources is provided in Table 1.

**Table 1:** Overview of search domains and illustrative search terms.

Search domain	Illustrative search terms	Data sources
Health literacy	*health literacy*, *Gesundheitskompetenz*, digital health literacy, organizational health literacy	PubMed, CINAHL, Embase, Google Scholar
Strategic health communication	health communication, strategic communication, communication values	PubMed, CINAHL, Embase
Normative values	transparency, trust, participation, equity, fairness, accountability	PubMed, Embase, Google Scholar
Organizational health literacy	organizational health literacy, HLHO, health literate organizations	PubMed, CINAHL, Google Scholar
German‐speaking context	Germany, Austria, Switzerland, D‐A‐CH, deutschsprachig	All sources

This was followed by thematically focused searches in Google Scholar, which does not reliably support complex Boolean operators. Short search strings were used, such as health literacy trust Germany, *Future Prospects Health Communication: Values in German‐Speaking Countries (Gesundheitskommunikation Werte deutschsprachig)*, organizational health literacy hospital, and adolescent health literacy social resources. In addition, theoretical sampling was undertaken to include conceptual works relating to communication values, institutional contexts, and systemic health literacy. As part of this sampling, key conceptual contributions from the German‐speaking health literacy literature were deliberately included [1, 27] to capture conceptual breadth and the German‐language system perspective.

Following duplicate removal, records were screened sequentially based on title and abstract relevance. Publications that addressed health literacy, strategic health communication, organizational health literacy, communication values, or the German‐speaking healthcare context were retained for full‐text assessment. Full texts were subsequently evaluated against the predefined inclusion criteria.

### 2.3 Rationale for a 15‑Year Search Window

The search window was restricted to the past 15 years to capture contemporary developments in strategic health communication and health literacy, particularly those related to digitalization, organizational health literacy, and evolving communication environments. First, the digital transformation of healthcare has profoundly altered public and institutional communication practices, with health information increasingly delivered through digital and platform‐based environments [13, 28]. The growing importance of social media, digital platforms, and emerging actors such as influencers has substantially changed how health information is distributed, accessed, and evaluated [13, 29]. Second, recent developments in artificial intelligence and algorithmically mediated information environments have further influenced how individuals search for, select, and interpret health information. Third, this period has been characterized by major conceptual developments in health literacy, including the emergence of digital and organizational health literacy and the development of measurement and implementation frameworks through international initiatives such as M‐POHL [1, 6, 22]. Finally, the COVID‐19 pandemic highlighted the importance of trust, transparency, risk communication, and the management of misinformation, thereby reinforcing the relevance of a contemporary time frame for studying strategic health communication and health literacy [1, 30, 31].

Restricting the search to 15 years ensures that the synthesis adequately captures current developments in the communication landscape and remains aligned with the contemporary relevance of values such as transparency, trust, participation, fairness, and digital orientation. Older studies were not included because their communicative environments, such as pre‑AI information architectures, lower levels of digitization in healthcare, and the absence of social‑media dynamics, no longer correspond to present‑day conditions and are therefore only partially interpretable.

### 2.4 Analysis and Synthesis

The analysis proceeded in two stages. In the first stage, all identified publications were assigned to the macro, meso, or micro level and their value‐related content was extracted. For the purposes of this study, values are defined as normative principles that orient communication, justify decisions, and shape expectations regarding legitimate, appropriate, or desirable practices. This includes both explicit values, such as transparency, trust, participation, fairness, user‐centredness, and equity, as well as implicit value dimensions that can be inferred from organizational routines, structures, discursive patterns, or user experiences.

The extraction process was guided by an inclusive understanding of values and did not rely on a rigid predefined taxonomy. Instead, values were identified inductively, drawing on established conceptual categories in strategic communication, public administration ethics, and the international health literacy discourse. This allowed the analysis to incorporate values documented in policy frameworks (e.g., accountability, inclusion), in organizational health literacy models (e.g., accessibility, responsiveness), and in interpersonal communication research (e.g., respect, emotional safety). Publications were included if they contained either explicit value statements or implied normative orientations relevant for communication and health literacy.

In the second stage, the extracted values were synthesized using theoretical triangulation. This synthesis drew on concepts from strategic communication science, organizational theories of organizational health literacy, and the contemporary multidimensional HL framework. The aim was to identify recurrent value configurations and determine their relevance for strengthening functional, interactive, and critical HL, as well as digital and organizational HL. Particular attention was paid to tensions between communicative aspirations and their practical enactment and to understanding how values are expressed, operationalized, negotiated, or undermined across different system levels. To enhance transparency and traceability, the findings of the synthesis are summarized in Table 2 (“Evidence‐Informed Value Configurations Across Macro‐, Meso‐, and Micro‐Levels”), which presents the dominant value configurations, their empirical grounding, and their relevance for health literacy.

**Table 2:** Evidence‐informed value configurations across macro‐, meso‐, and micro‐levels.

Step	Analytical activity	Output
1	Study classification	Macro, meso, micro level
2	Extraction of value‐related content	Explicit and implicit values
3	Cross‐study comparison	Recurring value patterns
4	Theoretical triangulation	Five value configurations
5	Health Literacy mapping	Functional, interactive, critical, digital, organizational Health Literacy

## 3 Results

The systematic literature search on the databases led to a result of 264 articles which were included in the subsequent screening. Following full‐text screening, 15 articles met the inclusion criteria and were included in the analysis [7, 13, 22, 24, 25, 28, 29, 30, 31, 32, 33, 34, 35, 36, 37]. Figure 1 provides an overview of the search workflow.

**Figure 1:**
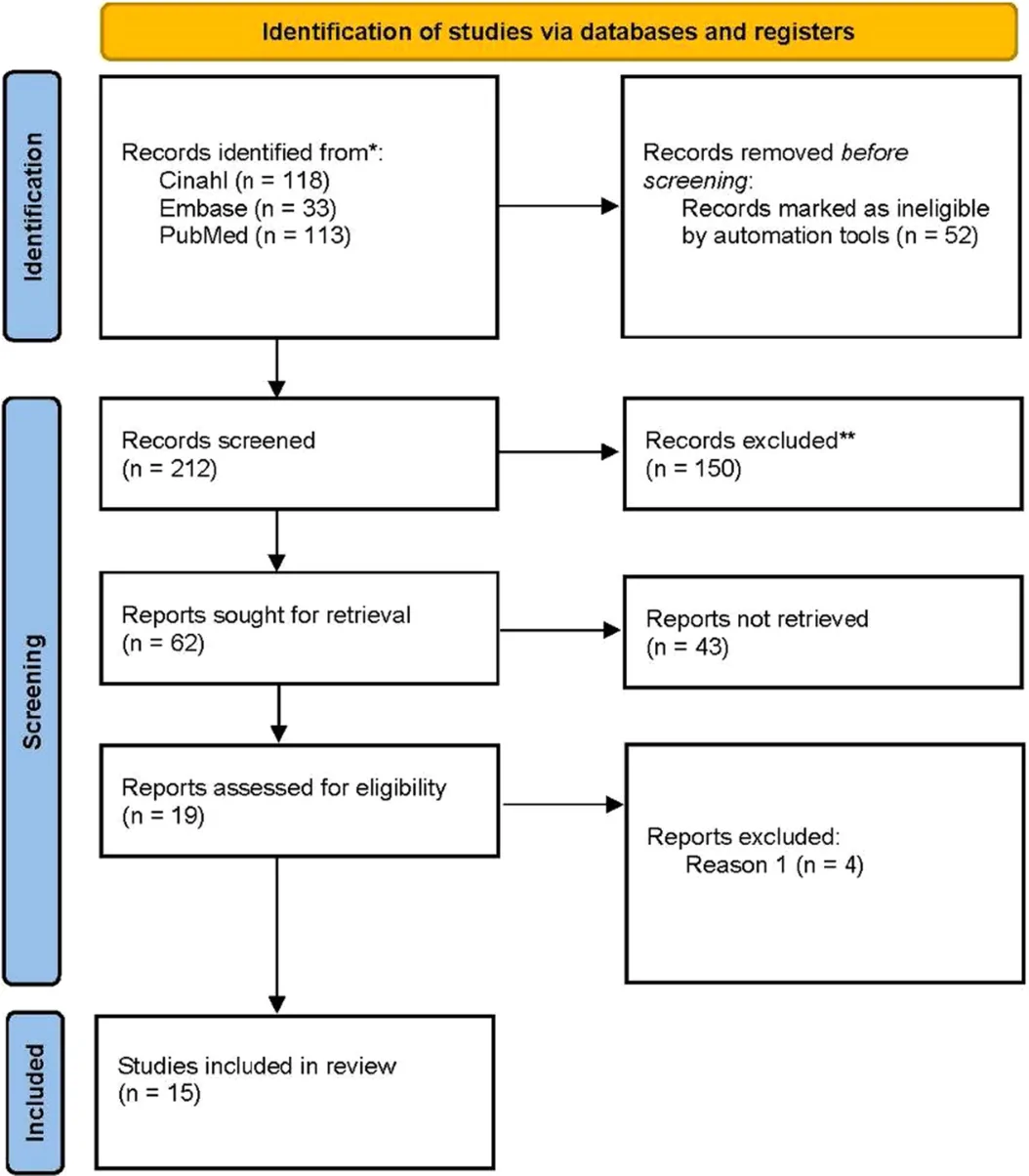
PRISMA flow diagram.

### 3.1 Synthesis Across the 15 Studies

Across the 15 included studies, a coherent value‐oriented pattern emerges that spans clinical care, education, digital health information, public‐health communication, vulnerable populations, and social media environments. The systematic categorization of findings into macro, meso and micro levels, followed by theory‐informed integration, reveals five overarching value configurations that shape both the conceptualization and the promotion of HL in structural ways. Table 2 provides an overview of the evidence‐informed value configurations identified across macro‐, meso‐, and micro‐levels. Table 2 and Figure 2 provide an overview of the systemic value architecture across macro, meso, and micro levels.

**Figure 2:**
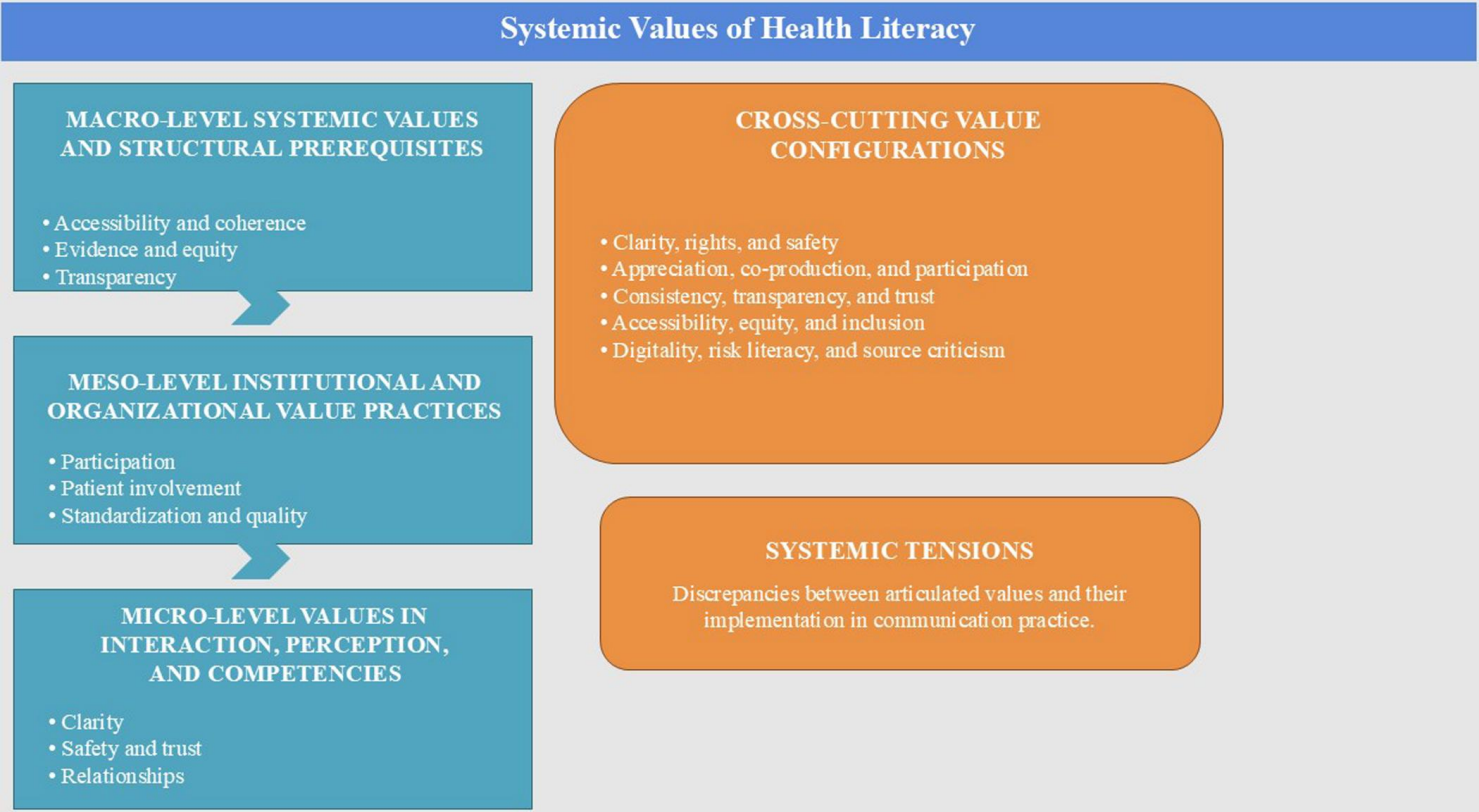
Systemic values of health literacy.

The value configurations reported below represent inductive and interpretive syntheses of recurrent themes identified across multiple studies. Where possible, explicit value statements reported by the original studies were retained; otherwise, broader value dimensions were derived from recurring communication characteristics and outcomes. Consequently, the value categories presented in this review should be understood as analytical interpretations rather than predefined classifications used by the original studies.

### 3.2 Macro Level: Systemic Values and Structural Prerequisites

At the macro level, the studies converge on a recognizable set of norms that structure the systemic role of health literacy within health systems, information ecologies, and public communication regimes. In this context, norms are understood as collectively shared and institutionally anchored expectations regarding appropriate communicative conduct, while values refer to the underlying normative principles that justify and motivate these expectations. This alignment becomes visible in research on professional HL competencies, organizational HL standards, and national HL strategies, all of which articulate expectations regarding clarity, accessibility, and user‑centredness as structural requirements for a health‑literate system [7, 22, 32]. Research on governmental crisis communication in Switzerland shows that citizens evaluate communication quality according to dimensions such as honesty, clarity, relevance, accuracy, consistency, and empathy. These dimensions were interpreted within the present synthesis as expressions of broader values including transparency (honesty, clarity, accuracy), accountability (relevance and responsiveness), and public orientation (empathy and consistency) [30, 31]. Equity, accessibility, and population‑level fairness also feature prominently at the macro level. Although population data on HL reflect individual abilities, they function empirically as system‑level indicators of structural inequities. A Swiss population‐based study of older adults demonstrated that individuals with multiple chronic conditions showed significantly lower HL across domains such as accessing, understanding, processing, and applying health information, with particularly pronounced deficits in health‐promotion tasks [37].

These findings do not describe micro‑level interactions but instead reveal how HL inequalities mirror structural gradients in morbidity, vulnerability, and access to resources, thereby highlighting the systemic relevance of equity and distributive fairness in health‑literate societies [37]. Studies on the digital health information landscape further indicate that commitments to transparency and accessibility are challenged by structural obstacles. A large‐scale computational analysis of the German Health Web shows that nearly 90% of high‐visibility health webpages are difficult or very difficult to read, suggesting a systemic failure to provide clear and usable information despite technically correct vocabulary [13]. Additional work on misinformation ecologies during COVID‐19 confirms that complexity, inconsistency and digitally mediated noise pose significant barriers to public understanding and appraisal [28].

### 3.3 Meso Level: Institutional and Organizational Value Practices

At the meso level, organizations are repeatedly portrayed as crucial translators of normative expectations into communication practices. Research on hospitals and long‐term care facilities highlights the importance of quality, standardization, and organizational responsibility, for example through the use of the Health‐Literate Health Care Organization 10‐item Questionnaire (HLHO‐10) and international organizational HL indicators, both of which emphasize navigation support, transparency, and patient‐centred communication as institutional responsibilities [7, 22]. These findings are consistent with the broader Health‐Literate Health Care Organization (HLHO) framework, which conceptualizes organizational health literacy as a set of institutional attributes that facilitate patients' navigation, understanding, and use of health information and services. In particular, the HLHO‐10 operationalizes key dimensions such as leadership commitment, workforce preparation, communication practices, and patient involvement [21]. Complementing this organizational perspective, the Health Literacy‐Sensitive Communication (HL‐COM) scale captures patients' perceptions of health literacy‐sensitive communication and highlights the importance of understandable information, supportive interactions, and responsiveness to patients' needs [20]. Together, these instruments reinforce the relevance of participation, patient‐centred communication, and organizational responsiveness as core components of organizational health literacy.

Studies on automated plain‐language patient letters demonstrate that organizational text governance can improve HL outcomes, reduce the prevalence of inadequate HL and increase perceived clarity and usefulness in clinical discharge communication [36].

At the same time, the meso level exhibits a pronounced gap between normative aspirations and organizational practice. Participation, co‐design, and patient involvement are widely endorsed in organizational health literacy frameworks and communication guidelines but remain only partially implemented in routine healthcare, educational, and digital settings [7, 22, 24, 29]. Studies further indicate that organizational constraints, such as limited time, privacy requirements, and communication barriers may restrict the practical enactment of participation and inclusion [24, 33].

### 3.4 Micro Level: Values in Interactions, Perceptions, and Competencies

At the micro level, clarity, comprehensibility, and safety emerge as pivotal values in interpersonal encounters and individual HL practices. Evidence from clinical communication research indicates that plain language, teach‐back, chunk‐and‐check and visual support procedures align closely with the macro‐level values that predict trust and acceptance in public communication while simultaneously addressing the readability problems documented in the digital environment [13, 32, 34]. In parallel, trust, relationship quality and emotion emerge as the strongest individual‐level predictors of HL‐related outcomes. When governmental or clinical communication is perceived as honest, respectful and empathetic, trust increases and information avoidance decreases, while support for recommended behaviours and perceived safety also increases [8, 31, 34]. Finally, critical, digital, and evaluative competencies are decisive in online environments. Among children and adolescents in Germany, the largest gaps involve privacy protection, navigation, and appraisal of reliability, with younger age and lower family affluence representing salient risk factors. These findings indicate that digital HL must prioritise source criticism, privacy literacy, and navigational scaffolding [25, 29, 33].

### 3.5 Cross‐Cutting Value Configurations

The cross‐level synthesis presented in Table 3 illustrates how value orientations identified at the macro, meso, and micro levels converge into broader patterns of health literacy promotion. Building on this synthesis, five recurring value configurations can be identified across system levels and contexts. Clarity, safety and rights underpin functional HL in both clinical and digital environments and are reinforced by evidence on readability, patient comprehension and crisis communication [13, 31, 36]. Participation, appreciation, and co‐production drive interactive HL but remain under‐implemented in institutional practice, as documented across hospitals, schools and youth services [7, 22, 24]. Trust, transparency and consistency sustain critical HL and risk communication and are shown to statistically predict behaviour in crisis contexts [8, 31]. Inclusion, equity and accessibility frame HL as an instrument for reducing gradients by age, morbidity and socioeconomic status, as shown for older adults with multimorbidity and for people with disabilities [37, 38]. Digitality, risk literacy and source criticism position digital HL as a protective competence set in hyper‐complex, algorithmically mediated information environments, especially for adolescents and multimorbid older adults [25, 28, 29].

**Table 3:** Macro–meso–micro value map (evidence‐anchored).

Level	Dominant values	Illustrative findings
Macro	Rights, transparency, coherence; equity and access; governance and evidence	Honest, clear, empathetic government communication predicts trust and acceptance; consistency deficits increase perceived risk. System readability gap challenges transparency goals
Meso	Quality, standardization, participation, learning culture; hybrid channel stewardship	Editorial governance can reduce sentence‐complexity barriers; institutional channels correlate with better perceptions of transparency and relevance. Implementation of participation lags.
Micro	Clarity, trust, emotional safety; critical–digital appraisal	Plain language and teach‐back align with macro predictors of trust; adolescents face gaps in privacy, navigation and reliability appraisal with social‐gradient effects.

### 3.6 Systemic Tensions

Finally, participation is widely affirmed but remains inconsistently realised in routine clinical, educational, and digital communication settings, while similar gaps exist between the value of coherence and the volatility of crisis communication, between trust and platform dynamics, and between accessibility and persistent structural gradients [7, 22, 31].

## 4 Discussion

The findings of the present synthesis show that strategic health communication in German‐speaking countries operates within a field of tension shaped by normative expectations, organizational capacities, and individual resources. These factors directly influence people's ability to access, understand, appraise, and use health information and services, thereby shaping key health literacy outcomes. This is particularly evident in the digital information environment. A large‐scale analysis of the German‐language health web demonstrates that nearly nine out of ten high‐visibility health websites are difficult or very difficult to read, even though the vocabulary used is largely understandable for lay audiences [13]. This reveals a structural problem that constrains functional health literacy not only at the individual level but already at the systemic level. The result aligns with theoretical work suggesting that HL should be conceptualized as a match between demands and available resources rather than as an individual deficit [4].

At the individual level, a complementary pattern becomes visible. Older adults with multimorbidity show significantly lower HL scores, with the greatest limitations observed in accessing, understanding and applying health information, as well as in making decisions related to health promotion [37]. The results indicate that the increasing complexity of chronic care continuously heightens the demands placed on HL, thereby generating a dual vulnerability. This observation corresponds with European work describing HL as a relational and context‐sensitive process that depends on structural support [3].

Similar challenges are evident in the domain of digital health literacy. Studies conducted among children, adolescents, and young adults indicate substantial inequalities in digital health literacy, particularly regarding privacy protection, navigation, and the appraisal of information quality, with younger age and socioeconomic disadvantage representing important risk factors [25]. For schools and healthcare providers, these findings highlight the importance of systematically supporting navigational skills, privacy competence, and source appraisal in digital information environments [25, 33]. These findings highlight the importance of values such as protection, fairness and empowerment, which are central to contemporary theoretical models of critical and digital HL.

Clear value relations also appear in the domain of governmental communication. Swiss data show that trust, clarity, consistency, relevance and empathy are significantly associated with evaluations of crisis communication, with acceptance of public‐health measures and with institutional trust [31]. These findings suggest that values do not remain abstract guiding notions but function as concrete communicative impact factors. They are also consistent with Sørensen's [2] proposition that HL represents a connection between individual competencies and systemic communication demands.

At the meso level, organizations act as key intermediaries that translate normative expectations into dependable communication practices. Studies on organizational HL show that hospitals rate themselves moderately positively, yet substantial deficits persist in patient‐centred information development, multimodal communication and staff training [2, 7, 22]. These findings illustrate the gap between values articulated in policy and theory, such as transparency, participation and justice, and their practical enactment in institutional routines.

At the micro level, studies on clinical and educational communication demonstrate that practices such as plain language, teach‐back and chunk‐and‐check align directly with the values empirically shown to be effective, including clarity, honesty, responsiveness and empathy. These practices foster trust, reduce uncertainty and enhance the understanding and application of information [32, 34].

The evidence base, however, shows several limitations. Many empirical studies are cross‐sectional, which limits the ability to draw causal conclusions [13, 25, 31, 37]. Moreover, several measurement instruments rely on self‐report, which can introduce bias, particularly when behaviours are infrequent and result in missing data, as observed in the digitally oriented subscales [25]. The decision to restrict the search to the past 15 years represents a further limitation of this review. Although this time frame was selected to capture contemporary developments in strategic health communication, digital health literacy, organizational health literacy, and evolving information environments, relevant earlier contributions may have been excluded. Consequently, foundational literature addressing communication values, trust, participation, empowerment, and health literacy prior to 2010 may not be fully represented in the synthesis. This is particularly relevant because several key theoretical foundations of health literacy and patient‐centred communication were developed before the widespread digitalization of health information environments. As a result, the value configurations identified in this review should be interpreted primarily as reflections of contemporary communication conditions rather than as a comprehensive historical account of the field. However, the chosen time frame was considered appropriate given the substantial technological, organizational, and societal changes that have shaped health communication during the past decade and a half, including digital transformation, social media expansion, and the COVID‐19 pandemic. Future reviews may benefit from integrating both contemporary and foundational literature to explore continuities and changes in value‐oriented strategic health communication over time. In addition, future research should examine how historical conceptualizations of trust, participation, and empowerment continue to shape current value‐oriented health literacy strategies and communication practices.

Future research should make greater use of longitudinal and experimental designs to systematically link values, communication practices and HL outcomes, and should place greater emphasis on the implementation of organizational standards. Overall, the synthesis demonstrates that value‑oriented health communication can make a substantial contribution to more equitable information environments, more resilient organizations, and higher levels of functional, interactive and critical HL in the German‑speaking context.

## 5 Conclusion

This synthesis shows that strategic health communication in German ‐ speaking countries is shaped by value configurations that act across macro, meso and micro levels. Distinct patterns become visible. Transparency, coherence and the right to understand guide communication at the system level. Quality, standardization and participation structure communication in organizations. Clarity, trust, and emotional safety shape interpersonal communication and individual health literacy processes. Evidence from 15 studies indicates that gaps between these levels—such as unreadable digital health information, limited organizational capacity for participatory communication or unmet needs among vulnerable groups—restrict the development of functional, interactive, critical, and digital health literacy. At the same time, interventions that use plain language, teach back routines, multimodal information design, and value driven crisis communication show measurable gains in comprehension, trust and engagement. Overall, the findings highlight that health literacy is not an individual shortcoming but a relational and system‐dependent capability that requires communication environments aligned with core values. Strategic health communication contributes to health literacy by enabling individuals to access, understand, appraise, and use health information and services effectively. Consequently, value‐oriented communication constitutes an important prerequisite for informed decision‐making, meaningful participation, trust, and health equity. Strengthening these communicative environments is therefore essential for promoting health literacy and supporting equitable and sustainable health systems.

## Author Contributions

**Slavko Rogan:** conceptualization, methodology, writing – original draft, investigation, writing – review and editing, data curation, formal analysis. **Katja Schlegel:** writing – review and editing, methodology, investigation. **Eefje Luijckx:** writing – review and editing, data curation, formal analysis, methodology.

## Funding

The authors have nothing to report.

## Ethics Statement

The authors have nothing to report.

## Conflicts of Interest

The authors declare no conflicts of interest.

## Transparency Statement

The first author, Slavko Rogan, confirms that this manuscript provides an honest, accurate and transparent account of the study described, that no significant aspects of the study have been omitted, and that any deviations from the study protocol have been explained.

## Data Availability

The data exchange does not apply to this article, as no new data sets were created or analysed as part of this study.
